# An Answer to a Statement of a Certain Case of Apoplexy. Signed W. Crowfoot, Beccles, Sept. 12, 1801

**Published:** 1801-12

**Authors:** R. Langslow

**Affiliations:** Halesworth, Suffolk


IT is inculcated by Dr. Fothergill, in the paper before referred to, that the obvious caufe of Apoplexy, in the greater number of cafes, arifes from the ftomach, and therefore the affection in the head is fymptomatic; and in conformity with this doctrine, he fays, We are as fpeedily as poffible to endeavour, by all the means we can, to remove the load by emetics, purgatives, &c.

We need not, fays he, be under much reftraint in the ufe of thefe remedies, till thorough evacuations are procured; the ftimulus excited in the ftomach, and the room provided by a freer circulation, are almoft alike beneficial, and without diminifhing the patient's ftrength, make way for his recovery. I am of opinion, fays the fame gentleman, that we fhould probably render the moft effectual aid by endeavouring, in all cafes, to procure a plentiful difcharge from the ftomach and bowels, as by thefe revulfions the head is never more relieved from plenitude, and that without weakening or interruption of other efforts of Nature to relieve herfelf.

---

There was alfo another Note, containing the cafe of a Mrs. N. who was attacked with Apoplexy, and attended by Mr. D. who cured her by judicioufly adminiftering emetics, as ftated in this note by Mr. C. Vide Dr. L's comment on this note.

---

*An ANSWER to a STATEMENT of a certain Case of APOPLEXY.* Signed W. CROWFOOT, *Beccles*, *Sept.* 12, 1801.

ON Wednefday, the 26th of Auguft, 1801, between feven and eight o'clock, P. M. I received a letter from a gentleman, ftating that his wife, a lady of great refpectability and confequence, was feized with an apoplectic fit, and requefting my attendance with all poffible fpeed. Upon being introduced into the parlour, I found the hufband, his brother, fon, daughter, and another gentleman whom I did not know, in the room. The hufband of the lady defired me to fee her inftantly, and report to *him* my opinion of her cafe. I obeyed, and found a young a young furgeon, Mr. P. in her room, who related to me all the fymptoms which had exifted upon his arrival two or three hours before, and his reafons for deeming the *attack apoplexy*; that he had confequently bled her immediately, and applied two large blifters to the fuperior part of the arms. His ftatement of the cafe and fymptoms, fuppofing them correct, convinced me *he was right* in his conjecture, and I approved of what he had done. He then informed me he had alfo fent for an emetic; I inftantly told him that I did not approve of emetics in Apoplexy, becaufe I confidered the difeafe as *always* arifing from compreffion of the brain; and in this cafe it muft be produced either by extravafation, exudation, or effufion; and as the act of vomiting tended to force the blood more violently into the head, or impede its return from thence, it would of courfe rather increafe than diminifh *the cause* of the difeafe. He was inftantly convinced of the propriety of my reafoning, and relinquifhed the idea. Mr. P. now afked me if I had feen Mr. Crowfoot? I told him I did not know Mr. C.; that there was fome ftranger in the parlour, but whether that was Mr. C. I could not fay. I now returned, and informed the hufband of the lady that I believed Mr. P. *right in his opinion* that the attack was apoplectic, and that there was, or had been, extravafation, which had been the caufe of it; that I highly approved of the bleeding her, and the application of the blifters.* Underftanding, however, that it was known that the exhibition of an emetic was in contemplation, *and my having now* forbidden it, I thought it my duty to explain my motives for fo doing, in juftice both to myfelf *and Mr. P.* who, as yet, I confidered as the *sole* propofer of it. I therefore faid, that though there was confiderable naufea and ficknefs at the ftomach, I confidered this fymptom as fymptomatic, or an effect of the compreffion of the brain, and not as a caufe or arifing from any foulnefs of the ftomach; I therefore thought that vomiting could not poffibly do good, but, on the contrary, might do *great injury*, by forcing more blood or ferous fluid from the veffels of the head, and thereby increafe the compreffion of the brain.† Inftead of the emetic I fhould therefore recommend leeches to the temples, which would ftill more unload the neighbouring veffels, and not tend fo much to debilitate the general fyftem as taking more blood from the arm would do. I was *thus proceeding*, when

* Not from their ftimulating or revulfionary powers, but from their tendency to take off or diminifh the hæmorrhagic difpofition or diathefis.

† No one, I believe, would think of an emetic to remove the naufea or ficknefs induced by a fractured fkull, combined with depreffion. And this is a cafe of apoplexy, though produced by accident or violence.

when the ftranger arofe, and obferved to the gentleman I was addreffing, that he fuppofed there was no farther neceffity for his remaining, and that he fhould therefore wifh to take his leave, particularly as he expected to be called to a labour. I inftantly requefted to know if that gentleman was Mr. C.? and being anfwered in the affirmative, I obferved that we ought to have been introduced to each other; and then faid to Mr. C. that notwithftanding *his presence* might be difpenfed with, as there was another Surgeon in the houfe; yet as he, Mr. C. had been in the habit of attending upon the lady, it might be more agreeable *to her* for him to apply the leeches, &c. &c. and that, notwithftanding any private difputes or difagreements, it was our duty when met, to exert our joint efforts to preferve fo valuable a life as that of our patient. Mr. C. now returned to his feat, and a meffenger who had been difpatched for leeches, returned with two very weak ones. I thought it ufelefs to trouble the lady with an attempt to apply them, as the attempt would probably be unfuccefsful, and only teize and trouble her to no purpofe. It was then agreed upon to fend another meffenger early in the morning to Loweftoffe, for more and better leeches; and Mr. C. took leave politely, requefting of me to know what hour in the morning I would wifh him to return? I propofed nine o'clock, but hoped, at all events, he would not exceed ten; and Mr. C. civilly affured me I might depend upon his arriving between nine and ten o'clock.

I fhall now revert to our patient, who had been affuredly much relieved by the bleeding, but ftill complained of confiderable pain, ftricture, and oppreffion in the head, attended occafionally with flight ficknefs and naufea. I thought it moft prudent, during this interval, to procure an evacuation from the inteftines, and ordered an opening mixture to be fent by Mr. Primrofe, and to be adminiftered in fmall dofes frequently repeated (to avoid, if poffible, exciting the action of vomiting) till the defired effect was produced. I then retired to bed, about twelve o'clock, with directions to be called again upon the acceffion, or farther increafe, of unpleafant fymptoms; I was not however difturbed till fix o'clock, A. M. when I was requefted to fee the lady inftantly, for that fhe complained of much greater pain, fullnefs, and ftricture in the head, together with an increafe of naufea. However, as the opening medicine had now juft fucceeded, and as the pulfe was tolerably free, I determined to wait the arrival of the leeches and Mr. C. rather than weaken the fyftem by taking more blood from the arm, unlefs the fymptoms of compreffion fhould ftill more advance, and compel me to bleed again from the arm, or temporal artery, which was my determination to do, if we did not fucceed in procuring ing leeches, in order to obviate a greater evil, namely, the increafe of compreffion by farther effufion upon the brain; fortunately Mr. C. and eight or nine good leeches arrived, foon after nine o'clock, and four or five were inftantly applied by Mr. C. with fuch fuccefs, that a very confiderable *evacuation* of blood was produced, with an immediate alleviation of the pain, ftricture, and fenfe of oppreffion in the head.

My apprehenfions of farther effufion upon the brain were therefore now much abated; I wrote a prefcription for fome faline draughts to be exhibited every fix hours, and I left my patient with great hopes of recovery. But before my departure, Mr. C. and myfelf agreed to meet again upon the following day, Friday, 28th of Auguft, at twelve o'clock. I was, however, unfortunately detained by another very urgent profeffional call, which prevented my arriving till half paft one o'clock, and I deemed it neceffary to apologize to Mr. C. for having caufed him to await my coming an hour and half. We found the patient much better, and without any fymptoms of frefh effufion, and the pain and oppreffion of the head were much abated, and I had reafon to believe the principal part of the effufed fluid was now *re-abforbed.* Upon our return to the parlour this day, a fhort altercation took place between myfelf and Mr. C. upon the nature and caufe of Apoplexy, in confequence of his advancing an opinion that no extravafation or exudation had taken place in the cafe of this lady. This converfation I fhall introduce with comments, when I come to the examination of his *own* ftatement of the cafe, after I have finifhed my prefent narrative. Our patient being now in a profperous way, I did not deem it neceffary to fee her on the following day, but left directions with Mr. P. to apprize me immediately of any return of the bad fymptoms, and directed him on fuch an emergency how to act till I fhould arrive. I, however, promifed at all events to vifit the lady on Sunday, the 30th of Auguft, and on my arrival that day, was informed by the gentleman of the houfe, that in confequence of his lady being fo much better, he had written to Mr. C. to difpenfe with his attendance upon that day, as my prefcription, he faid, could as conveniently be fent to Beccles to be prepared, as if Mr. C. had himfelf attended to convey them. However, my prefcription was as ufual forwarded to Beccles. Appearances were now fo favorable, I thought my attendance might with fafety be difpenfed with till Wednefday, the 2d of September, when I again promifed to attend; and on my arrival upon that day, I found our patient had experienced during my abfence, two or three periodical attacks of pyrexia, or febrile paroxyfms; upon which I deemed it neceffary to order the bark, which prefcription tion was difpatched to Beccles. And on account of the periodical attack of fever, I engaged to fee the lady again on Friday, the 4th of September, after laying down the neceffary plan of proceeding till I fhould return. And on that day, the 4th of September, I had the fatisfaction to find that my prefcription had fucceeded in preventing any frefh acceffion of fever, I therefore directed a continuation of the fame medicine, though not fo frequent, till Sunday the 6th of September, when I promifed again to attend. On my arrival this day, I found that the only marks of remaining difeafe were debility and general relaxation, for the removal of which, I directed a chalybeate combined with bark as a tonic, to be adminiftered twice or thrice a day, and confidered it as unneceffary to attend again before Wednefday, the 9th of September. But from this time little change appeared to be indicated in the mode of treatment, as fhe gradually recovered ftrength, and is at this period, I believe, in more perfect health than fhe had been for feveral weeks previous to her attack.

After this candid ftatement of the leading particulars in this bufinefs, judge of my aftonifhment when I was informed for the *first time*, upon vifiting B. laft Thurfday, *the* 23d *instant*, September, by the hufband of the lady, that Mr. C. confidered himfelf ill treated by me,* and that he had drawn up a ftatement of the cafe, but that the gentleman had refufed to perufe it, without Mr. Crowfoot would confent to let me fee it; this was at length acceded to, and this ftatement was fent to Mr. Primrofe to be exhibited to me. Upon reading it, I was aftonifhed beyond meafure, as from what I had heard of Mr. C. I had confidered him as a man of liberal education, both medical and otherwife; how far this conjecture of mine was well founded, I fhall fubmit to the opinion of thofe medical readers who are qualified

* Is an apothecary *ill treated* by a phyfician, becaufe the latter, unfortunately for the medical reputation of the former, happens to differ from him in opinion upon the utility of emetics in apoplexy, and poffeffes too much integrity to hazard the life of his patient, out of etiquette or compliment to him, on account of his having perhaps rafhly advifed the ufe of a very equivocal, if not truly dangerous remedy, in fuch a difeafe? Perhaps the world may be inclined to deem the attempt of the apothecary to oppofe the opinion of the phyfician, a much more ferious caufe of offence to the latter, than what the former could poffibly have received from the refiftance he experienced to the ufe of his emetic. Indeed, I have heard it ftated by fome, that they confidered the attempt of Mr. C. to contend with the opinion of the phyfician as an act bordering upon arrogance; to fay the beft of it, the meafure was affuredly indelicate, indifcreet, and impolitic; and it is more than probable, Mr. C. will think fo too, before he finifhes reading this anfwer to his ftatement of the cafe.

qualified to judge; but if his ſkill in the diagnoſtic and treatment of other diſeaſes, is no greater than what he appears to poſſeſs from this ſtatement, in regard to the cauſes, nature, and treatment of apoplexy, I am truly ſorry to have made this diſcovery, and ſtill more ſo that he, by his ſingular conduct in this buſineſs, ſo unwiſely and indiſcreetly compels me to make it public, and that merely to vindicate my own conduct and ſkill; for notwithſtanding I clearly diſcovered by converſation, at our *first interview*, that Mr. C's ideas of the cauſe and nature of apoplexy were extremely incorrect, yet I ſtudiouſly and delicately avoided to drop a hint of this diſcovery to the friends of the patient, leſt I ſhould injure the profeſſional reputation of Mr. C.; and this diſcovery would have been buried in oblivion, had not Mr. C. in his ſtatement, called upon me for a defence and juſtification of the opinions I had advanced; and though by many it may be deemed derogatory for a phyſician to condeſcend to contend, on *such an occassion*, with an apothecary, yet in this particular caſe, having had *those opinions* honoured by the ſupport and approbation of the family, I feel it incumbent upon me, for *their* juſtification, in having given a preference to my judgment, as well as for their ſatisfaction, to comment upon, and inveſtigate the merits of that ſtatement Mr. Crowfoot has thought proper to circulate.

Nemo mortalium omnibus Horis ſapit.

R. L.

Mr. C. obſerves on this caſe, that I declared that conſiderable extravaſation upon the brain exiſted;* and that the ſickneſs being the effect of the compreſſion, from the ſympathy which exiſted between the ſtomach and the brain, an emetic, as propoſed, could not poſſibly relieve this ſymptom, but in all probability aggravate it, by adding to the cauſe, and increaſe the extravaſation in the brain by forcing the blood with violence into the veſſels of the head, or preventing its return from thence, in the action of vomiting, and thus add to the original cauſe of the diſeaſe. He now ſtates his concealment from my knowledge as extremely irkſome, being made a witneſs to converſation in which his conduct was concerned. I can aſſure Mr. C. I did not then know that he had either ordered or *approved* the emetic; neither could I have ſuppoſed it poſſible an experienced practitioner of ſome celebrity, could have ventur-

* That the cauſe of apoplexy is always compreſſion of the brain from ſome cauſe, ſee Hoffman, (the father of modern practice in medicine) upon Apoplexy or Hæmorrhagia Cerebri, as he terms it; Boerhaave, Cullen, Fordyce, &c. &c. &c.

ed to recommend emetics in a diſeaſe he *had allowed to be Apoplexy.* In a note, Mr. C. obſerves, that Mr. P. during this time, was above ſtairs, and could have no opportunity of defending himſelf. What an inſinuation is this! What appellation does ſuch an inuendo as this note contains, merit? And allow me to aſk Mr. C. which of us has made the *moſt ſevere* and illiberal attack on the profeſſional character of Mr. P.; the phyſician, by explaining in his abſence (though with his approbation) his motives for rejecting the uſe of vomits in Apoplexy; or, the apothecary who *firſt apparently* approves and ſupports Mr. P's opinion, that this lady's caſe was Apoplexy? Nay, farther, approves the bleeding and bliſtering which had been had recourſe to on that *very principle!* Nay, himſelf recommends an emetic, upon the idea that *Dr. Fothergill had ſanctioned their uſe in Apoplexy!* And now comes forward to ſtate, that he admitted the caſe to be Apoplexy, to avoid *cavilling!* What a reproach is this then to the ſkill of Mr. P.! Nay, what ungenerous conduct to the patient and her friends! For if the caſe was not Apoplexy, but as Mr. C. *now* ſeems to inſinuate, a different diſeaſe, then, ſurely, his approving and recommending a mode of treatment particularly adapted to Apoplexy, and at the ſame time believing the diſeaſe a different one, what title does ſuch conduct deſerve? But, as the treatment proved ſucceſsful, is it not reaſonable to infer, that the firſt idea of the diſeaſe (and which idea ſuggeſted the treatment) was correct?

Mr. C. then proceeds to ſtate, that he announced himſelf in theſe words, That he was incapable of acting ſo vile a part as that of a ſpy. Now, I moſt ſolemnly aver, I never heard ſuch a declaration. To the beſt of my recollection, and it is alſo confirmed by thoſe who were preſent, his mode of announcement was this: "As Dr. L. is arrived, there can be no farther occaſion for my remaining here." And alſo, that he expected to be called to a labour. Upon which I aſked, if that gentleman was Mr. Crowfoot, &c. &c. as before ſtated in this Narrative. Neither can I remember any thing farther paſſing that was material, than my ſtating, that as he had been in the habit of attending this Lady as Apothecary, ſhe might probably approve of his applying the leeches in preference to Mr. P. who had not before attended her profeſſionally. This circumſtance, as ſhe was *now ſenſible*, was of ſome importance. When Mr. P. bled her, ſhe was *totally inſenſible*, and neither knew when it was done, or *by whom*; but being now in a *different ſtate*, his preſence might be uſeful on that account, though on *every other* we could certainly conveniently diſpenſe with it; and on ſuch an occaſion I thought all private diſputes ſhould be waved, and that we ſhould jointly exert our utmoſt efforts to preſerve

ſo fo valuable a life; that it was my rule, whenever my profeffional fkill was called into action, to exert it to the utmoft of my poor ability, even for an enemy.

This, as I recollect, was the exact purport of our converfation, though it appears, in SOME refpects, to *differ* from Mr. C's Statement. I now introduced the fubject of the leeches, with obferving, that they would more immediately empty the head, and thereby enable the abforbents more readily to take up the effufed fluid, and weaken the fyftem lefs than a farther general bleeding. Mr. C. approved of this doctrine, but *denied* the extravafation. All modern authors are *decided* upon this point,* that compreffion is the *only* caufe of Apoplexy and that produced by the effufion of blood or ferum, and generally the former. Mr. C. then proceeds to ftate, that in his judgment, if confiderable extravafation had taken place, the moft formidable fymptoms muft have arifen; I afk Mr. C. what *more* formidable could arife than did in this cafe? The vital and animal functions, except thofe of the heart and refpiration, were *completely arrested*; fhe was totally *senseless*, and would have fallen to the floor if fhe had not been fupported; her refpiration was laborious, and her jaw fell; and thefe alarming appearances continued a confiderable time, and did not in the leaft abate till about twenty minutes, or perhaps half an hour, after Mr. P. had bled her. If thefe fymptoms were not fufficiently formidable to *indicate Apoplexy*, I fhould fuppofe Mr. C. would not be inclined to admit of any thing fhort of *death itself* to be fufficiently fo. Indeed, he foon after intimates, that if extravafation had really exifted in this cafe, the lady would not have been in a ftate to require our help; or, in other words, that whenever extravafation exifted in the brain, it was immediately fatal.†

But,

---

* Except in cafes of fractured fkull or tumours forming in the head. In a converfation I have fince had with the truly intelligent and fcientific Phyfician, and Lecturer, Dr. G. Fordyce, he affured me, he believed apoplexy was always produced by compreffion, and that compreffion rarely, if ever, was produced by any other caufe than *extravasation of blood*, and feldom if ever from ferum only.

† Mr. C. appears from his mode of reafoning upon extravafation on the brain, to have no idea of an abforbing power exifting in the animal œconomy. It is however, extraordinary if he fhould be *now* to learn that every furface of the body, both internal and external, is fupplied with a fet of veffels for *this express purpose*, called lymphatics; and notwithftanding thefe lymphatics have not as yet been injected in the brain, the beft phyfiologifts are of opinion, that this neceffary office may be performed *there*, by the orifices of the inhaling veins. Indeed, if fuch a power did not exift in the head, Mr. C's ideas of fpeedy diffolution, in *all cases* of extravafation on the brain, or

 apoplexy,

But, fays Mr. C. there was no mark of paralyfis. Is Mr. C. then yet to learn, that probably half the cafes of Apoplexy which occur, do not terminate in paralyfis? If he will refer to the Works of the great, I had almoft faid the immortal Cullen, he would fee this opinion of mine *completely confirmed.*

Mr. C. then ftates, that on Thurfday the 27th fhe had paffed a good night. Why then was I called to her relief at fix o'clock, when *frefh symptoms* of effufion began to appear, but which were alleviated by the evacuations which at *this period* were produced from the inteftines? And though Mr. C. fays fhe was this morning manifeftly mended, yet thofe about her will inform Mr. C. that fhe was by no means fo well this morning as fhe was when her hufband left her to retire to bed the night before, though fhe was immediately and *manifestly relieved* by the application of the leeches. On our meeting the following day, the 28th, Mr. C. was ftill fo much attached to his favourite idea, that extravafation was not neceffary for the production of Apoplexy, that he again renewed the argument; when, in order to put a ftop to a converfation that I confidered fo contrary to the eftablifhed doctrines of the day, I affured him it was my firm belief that *no case of real Apoplexy* could poffibly occur without compreffion; and that when the fkull was not injured, the compreffion was always produced either by extravafation, exudation, or effufion, in *every instance*, when it amounted to an abolition of the fenfes, and that the patient fell to the ground. He then afked me if I had feen a cafe of Epilepfy? I could not refift fmiling at this queftion; and obferved, that I hoped he was capable of diftinguifhing between Apoplexy and Epilepfy: We were not alluding to Epilepfy; and furely Mr. C. could not fuppofe I meant to affert, that in every cafe of infenfibility there muft neceffarily be extravafation.— Epilepfy, fyncope from lofs of blood, hyfteria, delirium in fever, and many other caufes of abolition of fenfe, may arife where no extravafation on the brain exifts. But what I contend for is this, that whenever *Apoplexy occurs* to the extent before ftated, and there is no fracture or tumour, then I affert, that extravafation, exudation, or effufion upon the brain *always does exist.*

Mr. C. next ftates, that he was aftonifhed to hear me declare fo pofitively, after a firft fight of the patient, that there was apoplexy, would be *well founded.* But thanks to an all-wife and bountiful Difpofer and Director of thefe things, no part of the animal machine is void in this very neceffary appendage to the vafcular fyftem, without the aid of which fudden death would be hourly, nay minutely occuring, and the univerfe be fpeedily depopulated.

was confiderable extravafation. Now, I believe, I did *not* fay *confiderable*; though, if I did, the fymptoms which Mr. P. told me exifted till *after she was bled*, were fully equal to fupport *such an opinion*. But, as I find none of the parties prefent except Mr. C. heard the word *considerable* made ufe of, *is it not possible* he may be miftaken?—I do recollect ufing the words extravafation and exudation; but Mr. C. declares thefe two words convey a diftinction without a difference. But here Mr. C. unfortunately again evinces a great want of difcrimination, and I fhall trouble myfelf to explain it. The term extravafation ought to be ufed when the blood or fluid efcapes through a rupture in the coats of the veffels; and the term exudation when the fluid is forced through the exhalants. In the firft, red blood will be effufed; in the latter, ferum or lymph; and this forms the only true diftinction between fanguineous and ferous Apoplexy. A doctrine which certainly ought to be clearly defined and underftood by every practitioner, as many valuable lives, I am now convinced, have been loft by the want of a perfectly clear idea of this diftinction; by fuppofing that bleeding in the ferous Apoplexy is ufelefs, if not injurious.* But, in truth, the operation of bleeding is indifpenfably neceffary in Apoplexy, whether the compreffing fluid is blood or ferum, and, confequently, whether the Apoplexy is *sanguineous* or *serous*. And it is now my determination, from feeing the danger of defective difcrimination in *these cases*, the moment I have a little leifure, to write upon this fubject in fuch plain and intelligible terms, as to enable the young and moft inexperienced practitioner, when called upon in fuch emergencies, to act with promptnefs and decifion, and with confidence in himfelf till more experienced affiftance can be procured; as a few minutes delay, in fuch inftances, might prove fatal.

Mr. C. now ftates, that I faid the beft anatomifts were of different opinions. My declaration was, that the *best had differed* upon the fubject. I meant formerly; for I believe at the *present moment*, there is only one opinion on the fubject, except that of Mr. Crowfoot's, that *real Apoplexy* is caufed by compreffion of the brain, and always, when the fkull is *not* fractured, by the effufion of blood or ferum.

Mr. C. in his Notes, introduces a fuccefsful cafe, in fupport of his favourite doctrine, that extravafation is not the caufe of Apoplexy: That a lady attacked with Apoplexy, where the fymptoms

* From what I have lately heard from good authority, this was the cafe with the father of the prefent Lord Rous; who, had he been bled fooner, would affuredly have had a better chance of relief.

ſymptoms were exactly ſimilar to thoſe of this patient, to whoſe aid a Mr. D. was called, and that he *cured* this patient by *vomits*. But is it neceſſary to remind Mr. C. that ſucceſs is by no means a certain criterion of the propriety of a particular remedy, eſpecially when others of a leſs doubtful nature are uſed at the ſame time? Or that Nature in this, as well as in other critical ſituations, ſometimes happily triumphs over the *remedy* as well as the diſeaſe? Otherwiſe, how can we account for the number of miraculous cures, and hair-breadth eſcapes ſo frequently attributed to ſome of the moſt inſignificant if not injudicious means? In ſhort, may we not with propriety add, that in *this instance*, if vomiting was *solely* depended upon, that ſhe recovered in defiance of the Doctor, and that the vis medicatrix naturæ was too powerful to be ſubdued?

Upon the merits of this paper, allow me to aſk Mr. C. how the brain can be compreſſed by a foul ſtomach? Or does he believe that the action of vomiting will not determine more blood to the head? And does Mr. C. really think that ſtimulating the ſtomach by emetics will produce a *freer circulation* than emptying the *blood vessels themselves*? And farther, that plenitude is more expeditiouſly obviated by *vomiting* than *bleeding*? I can only ſay, that if the late Dr. F's other works and practice were upon a par with this paper on Apoplexy, he never would have gained that celebrity in his profeſſion he once poſſeſſed.

I have

I have now finiſhed my Critique upon Mr. C's Paper, and, I truſt, without either virulence, acrimony, or ill nature; at leaſt, it has been my anxious wiſh ſo to do, and to act with that liberality and candour, on this and every other occaſion, which is aſſuredly due from one profeſſor of the ſcience to another, be his reſpective department in that profeſſion what it may; and if by means of this little colliſion of ſentiment between Mr. C. and myſelf, a ſpark of real information ſhould unexpectedly be elicited, leading to a more ſcientific and rational plan of practice in Apoplexy, I ſhall feel myſelf much gratified, and amply rewarded for my trouble, by having contributed my mite towards the reduction of the long liſt of evils human nature is, alas! ſubjected to.

R. LANGSLOW.

*Haleſworth, Suffolk, Sept. 26, 1801.*

N. B. I did not ſee Mr. C's Statement till late in the day of the 23d inſtant, and had not a moment's reſpite from profeſſional fatigue till this day, (26 Sept. 1801.)

